# On the Utility of the Apothecaries Act

**Published:** 1813-05

**Authors:** J. Jones

**Affiliations:** Guisbro', North Riding of Yorkshire


### *To the Editors of the Medical and Physical Journal.*

GENTLEMEN,

I AM induced, through the medium of your truly-valuable Journal, to make a few remarks on the much-to-be-lamented disputes which at present exist amongst medical men, in consequence of the Apothecaries Bill, now before Parliament. The observations made by one of your correspondents in your last Number, who signs himself Salus Publica, call particularly for animadversion; more so, as they receive some sanction by finding a place in your publication. His harsh severity against such a numerous and respectable body of men as the surgeon-apothecaries are, can only be attributed to the paltry feelings of jealousy and selfishness, which, among mechanics, or the lower orders of society, are not uncommon; but that they should exist in a liberal and scientific profession, is greatly to be lamented. The observations of Censorinus appear to be dictated by similar principles; and I strongly suspect that these gentlemen are so much influenced by interested views, that they would gladly sacrifice the respectability of their profession to individual aggrandisement, and that from an erroneous belief that the physician is not consulted on all necessary occasions, would stamp the surgeon-apothecary with ignorance and ignominy; and would, no doubt, sincerely rejoice if every surgeon-apothecary were really the ignorant character racter they would insinuate, as then their *imaginary* opposition would not appear so formidable to them. From such men we are not to look for professional improvement; but I should advise them to recollect, that, when they endeavor to detract from the respectability of the surgeon-apothecary, they are at the same time lessening that of the physician, for we are all *membra ejusdem corporis.*

The duties of each department of the profession have been long defined by the sanction of custom. From the nature of things, it becomes indispensable for the surgeon-apothecary to prescribe in ordinary cases, and when there is danger or difficulty he calls in the physician; or, indeed, when the pecuniary circumstances of the patient will admit, he is consulted nearly as soon as the surgeon-apothecary; and this mode of proceeding, I conceive, every one will acknowledge to be most fully established. The object of the bill before parliament is to engage the legislature to fix such regulations and restraints as will effectually prevent ignorant pretenders from obtruding themselves into the profession, and imposing on the public; and to allow the well-informed surgeon-apothecary the remuneration which his education, industry, and utility, entitle him to.

As Salus Publica does not appear to be aware of the prerequisites for constituting a respectable surgeon-apothecary, I shall just mention to him the advantages which are generally considered necessary for him to possess, and which every one, before he commences the important duties of his profession, *ought to be forced by law to receive.* Salus Publica says, "as their system of education is cheaper," &c. &c.; in this I must beg to differ from him, without wishing to detract from the erudition and professional knowledge which every physician is supposed to possess; admitting, however, that for want of the interference of the legislature, there are characters of the most ignorant description practising in each branch of the profession. A young man intending to practise as surgeon-apothecary, in general pays a high premium for his indentures; having served his apprenticeship, he becomes student at some of the public hospitals in London, or one of the respectable Scotch universities. The time considered necessary to be spent at these medical seminaries is generally allowed to be two years; during which, they so far become proficient in the different branches of their profession, as to be enabled to pass a public examination; which having done, they receive the diploma of the Royal College of Surgeons, and forthwith commence practice, with the confidence which conscious knowledge naturally produces. We well know that it is only required for a student to be three three winters and a summer at Edinburgh or Glasgow, to entitle him to become a candidate for a diploma for Doctor Medicinæ. I do not say that all physicians confine themselves to the prescribed advantages; but, certainly, when these are all that are required by the College of Physicians, I conceive that Salus Publica has done injustice to the physician, in assuming such unbecoming superiority over the surgeon-apothecary; and to the latter, by endeavoring to detract from the respectability due to him. Every unprejudiced mind must rejoice at the fair prospect presented by the present bill in parliament for medical reform; and I am persuaded, that if medical gentlemen would be unanimous in promoting a measure which is so likely to place our profession in the dignified station which it ought to hold, they would secure to every department of it due honor and emolument. I am, Gentlemen,

Your most obedient Servant,

*Guisbro', North Riding of Yorkshire,* J. JONES.

*April* 8, 1813.

